# A white raven detected by imaging

**DOI:** 10.1007/s12471-015-0706-5

**Published:** 2015-06-02

**Authors:** L.H.B. Baur, W.M.J. Schreurs, H.R. van Leeuwen-Wintjes, C.L. Berendsen, R. Willems, R.A.G. Winkens, R. Vliegen, P. Theunissen, E.B. Gomez Garcia

**Affiliations:** 1 Department of Cardiology, Atrium—Orbis location Heerlen, Henri Dunantstraat 5, 6401 CX Heerlen, The Netherlands; 2 Maastricht University, Maastricht, The Netherlands; 3 Department of Nuclear Medicine, Orbis location Heerlen, Heerlen, The Netherlands; 4 Department of Internal Medicine and Endocinology, Atrium—Orbis location Heerlen, Heerlen, The Netherlands; 5 Department of Anaesthesiology, Atrium—Orbis location Heerlen, Heerlen, The Netherlands; 6 Department of Pathology, Atrium—Orbis location Heerlen, Heerlen, The Netherlands; 7 Department of Radiology, Atrium—Orbis location Heerlen, Heerlen, The Netherlands; 8 Department of Urology, Atrium—Orbis location Heerlen, Heerlen, The Netherlands; 9 School for Public Health and Primary Care, Maastricht University, Maastricht, The Netherlands; 10 Department of Clinical Genetics, University Hospital Maastricht, Maastricht, The Netherlands

**Keywords:** Phaeochromocytoma, Incidentaloma, MIBG scan, Hypertension, CT scan, Imaging

## Abstract

The purpose of this case report is to describe a rare case of a patient with a phaeochromocytoma with several cardiovascular complications, which can be attributed to the tumour. Detection of a phaeochromocytoma sometimes needs a ‘Sherlock Holmes spirit’ or simply time.

A 59-year-old man was admitted with a cerebral infarction. He showed atrial fibrillation, signs of heart failure and severe hypertension. The electrocardiogram showed atrial flutter with a fast ventricular rate. His echocardiogram showed a poor contracting left ventricle. During the follow-up, his blood pressure and cardiac function normalised and the rhythm returned to sinus rhythm. The coronary angiogram appeared normal. In 2013, an abdominal echo and computed tomography (CT) scan were performed because of abdominal complaints. This revealed a mass close to the right kidney (Fig. 1). The I-123 MIGB SPECT (Also known as Iodium 113-**metaiodobenzylguanidine**SPECT)scan showed pathological stacking of I-123 (Fig. 2). The diagnosis phaeochromocytoma was made. The tumour was surgically removed. Pathological examination revealed a benign phaeochromocytomas. DNA testing in the Clinical Genetics Department excluded hereditary causes. Phaeochromocytomas are frequently discovered by chance during a radiological examination [1]. Next to CT and magnetic resonance imaging, molecular imaging should be considered for analysis [2, 3].

**Fig. 1:**
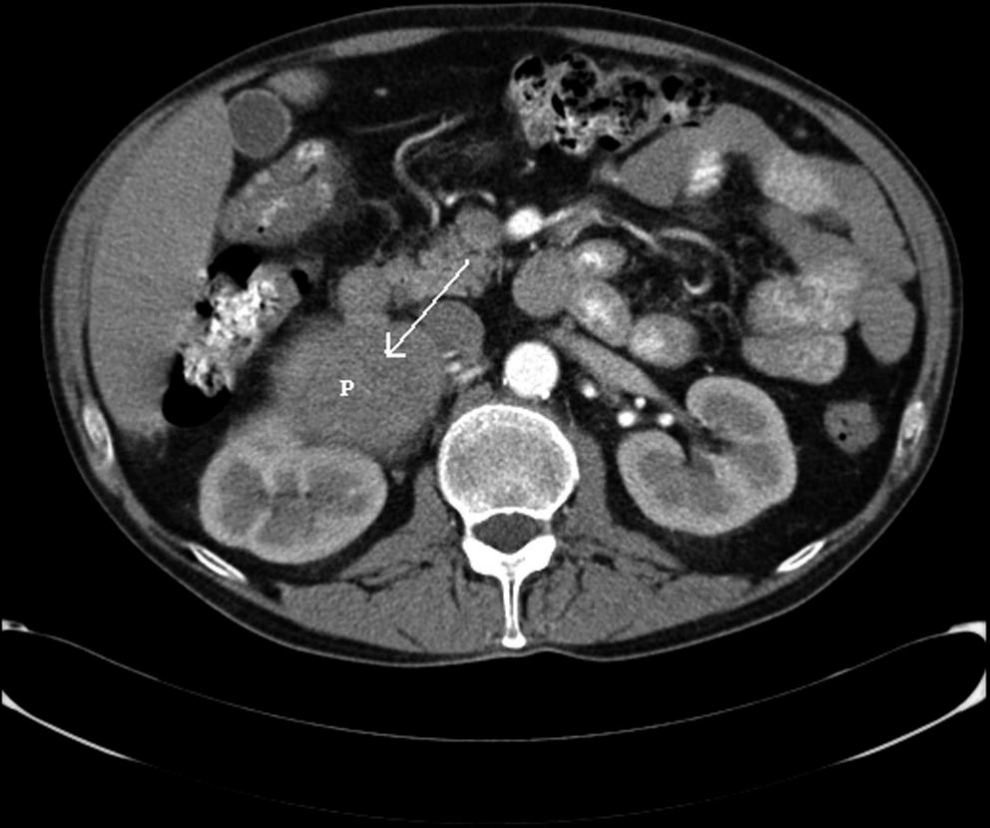
Abdominal computed tomographic scan showing a huge mass (*arrow*) starting from the right kidney

**Fig. 2:**
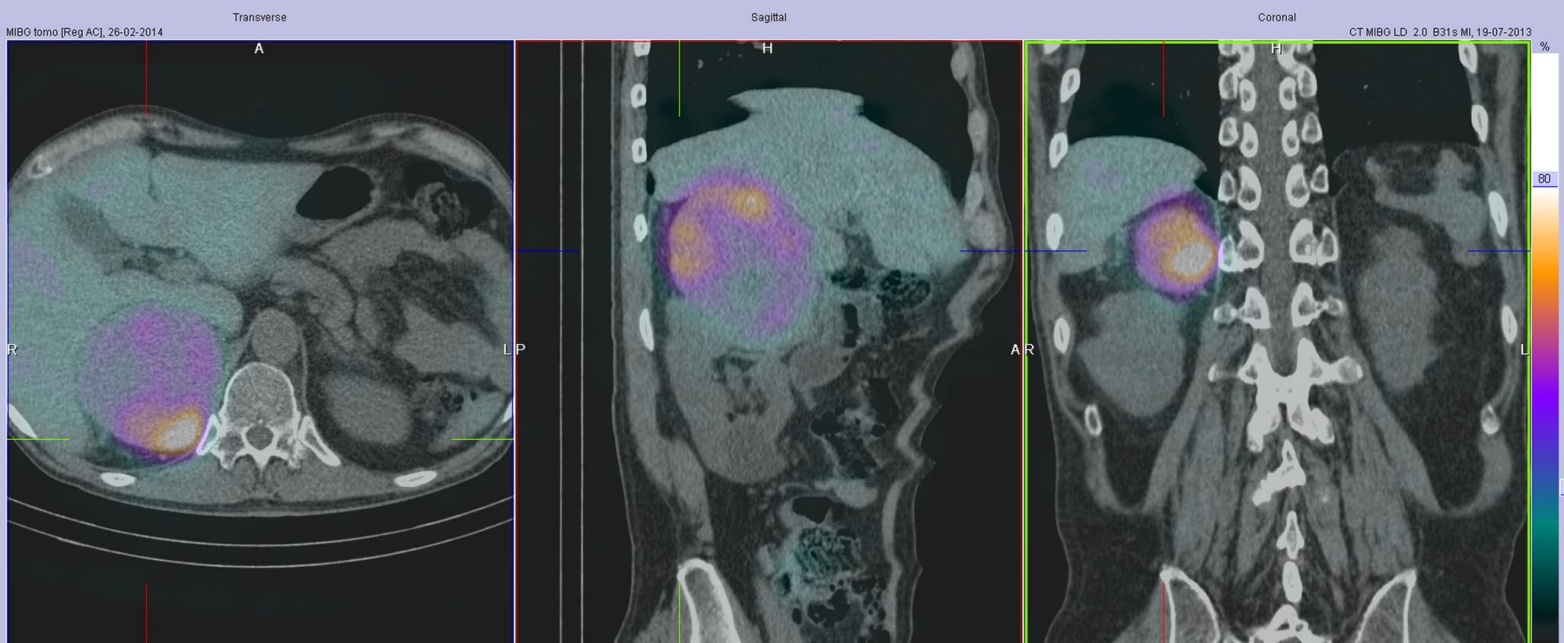
MIB SPECT scan of the thorax showing a large mass in the right upper abdomen

## Permission

The patient, whose disease is described, has given oral informed consent to publish the case report.

### Funding

None.

### Conflict of interest

None declared.
